# Clinical Application of Botulinum Toxin for Hemifacial Spasm

**DOI:** 10.3390/life13081760

**Published:** 2023-08-17

**Authors:** Chang-Kyu Park, Seung-Hoon Lim, Kwan Park

**Affiliations:** 1 Department of Neurosurgery, Kyung Hee University College of Medicine, Seoul 02447, Republic of Korea; changcha@khu.ac.kr (C.-K.P.); limshns03@gmail.com (S.-H.L.); 2 Department of Neurosurgery, Konkuk University Medical Center, Seoul 05030, Republic of Korea

**Keywords:** hemifacial spasm, botulinum toxin

## Abstract

Hemifacial spasm is typically caused by contact between the facial nerve and blood vessels. Microvascular decompression, a treatment that directly addresses this pathogenesis, is often considered the most effective treatment method. However, surgery is not immediately performed for patients at risk from the surgical treatment, or for those with an unclear diagnosis. In these instances, Botulinum toxin injection can help manage the patient’s symptoms. Numerous studies corroborate the effectiveness and safety of Botulinum toxin treatment, with large-scale studies indicating symptom control lasts, on average, around 15 weeks.

## 1. Introduction

Hemifacial spasm (HFS) is a neurological disorder characterized by involuntary contractions of muscles on one side of the face [1,2,3,4]. Its primary symptom involves repetitive, involuntary twitching or spasms of the facial muscles, typically initiating around the eye and potentially spreading to other muscles on the affected side. These spasms and twitching, which mainly impact the eye, cheek, and mouth muscles, are generally confined to one side of the face. Over time, these spasms tend to escalate, growing more frequent and intense; they might begin as occasional twitches and develop into sustained contractions. As the condition advances, the continual contractions can lead to facial asymmetry, with the muscles on the affected side becoming hyperactive and more prominent compared to the unaffected side. The resulting symptoms often complicate patients’ daily lives and interpersonal relationships, making most patients eager to pursue appropriate treatment.

The etiology of HFS is often associated with the compression or irritation of the facial nerve [1]. When this nerve is impacted, it can trigger the typical muscle contractions and spasms observed in HFS. The most prevalent cause of HFS is believed to be the compression of the facial nerve by a neighboring blood vessel, typically an artery. This condition creates an abnormal contact, or conflict, between the facial nerve and the blood vessel. Such contact can lead to irritation and demyelination of the facial nerve, ultimately causing the spasm [5,6,7].

Considering these etiologies, the most definitive treatment for HFS is microvascular decompression surgery, the outcomes of which are generally quite favorable [8,9]. However, in cases where the diagnosis is unclear, the patient refuses surgical treatment, or general anesthesia poses a risk, Botulinum toxin (BoNT) injections into the facial muscles serve as an alternative method for symptom control. This opinion article reviews the results of large-scale studies published on the effects of BoNT treatment on HFS, and describes injection sites, side effects, and future development potential.

## 2. Pharmacology of Botulinum Toxin

Botulinum toxin (BoNT) is a potent neurotoxin that is used for various medical and cosmetic purposes. The most commonly used form of botulinum toxin is botulinum toxin type A.

BoNT operates by blocking the release of acetylcholine, a neurotransmitter responsible for transmitting signals between nerves and muscles. It attaches to specific receptors on presynaptic nerve terminals, preventing the fusion of vesicles carrying acetylcholine with the cell membrane. This blockade inhibits the release of acetylcholine, effectively mitigating muscle contractions. BoNT is directly injected into the targeted muscles or tissues, where it is locally absorbed at the site of administration. The toxin remains largely localized near the injection site, demonstrating minimal systemic distribution. The effects of BoNT are temporary and reversible, typically lasting around three to four months. As nerve terminals regenerate and form new connections, muscle contractions gradually resume. BoNT is broken down enzymatically within the body, predominantly by proteases, and its metabolites are eliminated through regular metabolic pathways [2,10,11].

## 3. Injection Site of BoNT for Hemifacial Spasm

**Figure 1:**
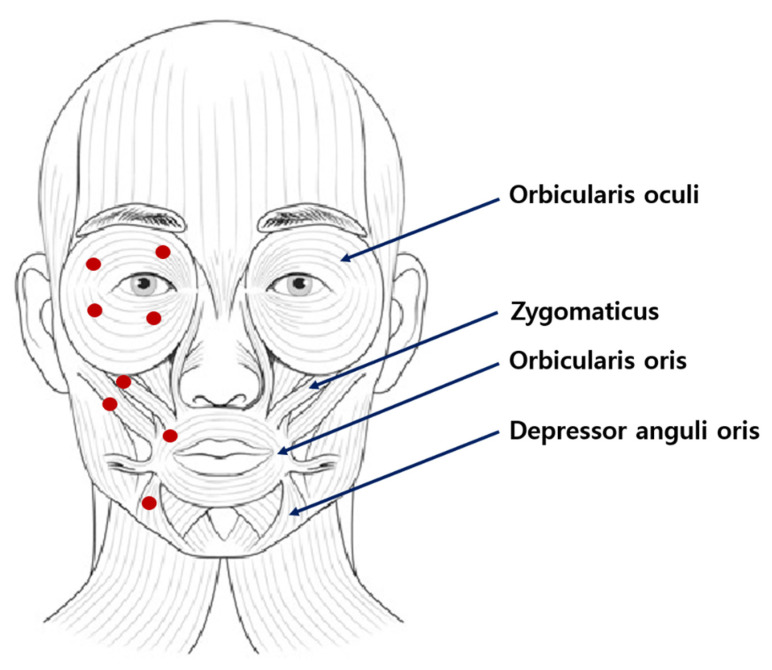
Most frequently treated muscles in hemifacial spasm, Red circle is a typical injection site.

When using BoNT for the treatment of hemifacial spasm, it is essential to follow practical guidelines to ensure safe and effective administration. Precise injection technique is crucial for optimal results, which requires identifying the specific muscles involved in the spasm and administering injections directly into those muscles. The dosage and injection sites may vary depending on individual needs, but commonly targeted muscles include the orbicularis oculi, zygomaticus, orbicularis oris muscles, and depressor anguli oris muscle [2,3,11,12] (Figure 1).

Orbicularis oculi muscle: This muscle surrounds the eye and is responsible for eyelid closure. Injections are typically given in the orbicularis oculi muscle to target eyelid spasms and reduce excessive blinking. Multiple injection points may be used along the upper and lower eyelids.

Zygomaticus muscle: The zygomaticus muscle is located in the cheek area and is responsible for facial expressions such as smiling. Injections in the zygomaticus muscle can help alleviate spasms and abnormal movements in the cheek region.

Orbicularis oris muscle: The orbicularis oris muscle is around the mouth and is involved in lip movements and expressions. Injections in this muscle can be beneficial for reducing involuntary contractions and twitching in the mouth area.

Depressor anguli oris muscle: This muscle is located at the corner of the mouth and is responsible for the downward movement of the lips. Injections in the depressor anguli oris muscle can help control abnormal movements and spasms in this area.

The dosage of BoNT varies depending on factors such as muscle size, severity of symptoms, and individual response. The initial dosage is often conservative, with subsequent adjustments made based on the patient’s response and tolerability, i.e., start with lower doses and gradually increase if necessary [2,3,11,12]. Currently, total doses recommended for HFS for each session should range accordingly: 10–34 unit for Onabotulinumtoxin A, 53–160 unit for Abobotulinumtoxin A, and 1250–9000 unit for Rimabotulinumtoxin B. The therapeutic effect begins at about 3–6 days after treatment and can persist for 2–3 months. Intervals of 3 months between injections are generally recommended [2,3,11,12]. Usual doses of Botulinum toxin A per site are summarized in Table 1.

## 4. Clinical Results of BoNT for HFS

BoNT is an approved treatment for HFS in many countries, including the United States and those in the European Union. The U.S. Food and Drug Administration approved BoNT for the treatment of various neurological disorders that cause muscle spasms, including HFS, in 1989. It has been in use for this condition for several decades now and is generally considered to be safe and effective.

BoNTs offer symptom relief without the adverse effects associated with surgery. Reports indicate that BoNT injections have improved the quality of life for those suffering from HFS. According to large-scale studies, the efficacy of BoNT ranges from 78% to 98.4%, with the mean duration of the effect being around 15 weeks [15,16,17,18]. The results from various large-scale studies involving over 100 patients are summarized in Table 2.

BoNT treatment schedules are typically set at 12-week intervals, based on the expected duration of the drug’s effect. However, in some cases, the effect may diminish more quickly, necessitating a shorter injection interval. Under these circumstances, a flexible adjustment in the BoNT injection dose might be required. Over the course of therapy, the standard doses of injected botulinum toxin have been observed to increase for most patients.

Most doctors initially administer small doses of BoNT due to concerns about potential side effects. Another factor is that as the disease progresses, symptoms can spread to other muscles, leading to an increase in the overall BoNT injection dose. One long-term follow-up study on BoNT therapy in patients with blepharospasm and hemifacial spasm found a higher average injection dose per visit in the final year compared to the first year of treatment. However, they reported that as the dosage increased, so did the duration of the BoNT effect in comparison to the initial injection [20].

In addition, a recent randomized, double-blind study evaluated the necessity of oral BoNT A injection in hemifacial spasm (HFS) patients [21]. A total of 60 patients were divided into two groups, receiving BoNT A either around the eye only or both the eye and mouth. The results showed similar improvement durations in both groups, with a higher mouth symptom improvement but also more side effects in the group receiving BoNT A around the mouth. The study concluded that mouth injection of BoNT A may not be necessary for HFS treatment.

## 5. Evaluation Scale for HFS

To assess the severity and impact of HFS on patients, several scales have been developed. This manuscript outlines the point rating scale, visual analog scale (VAS), Chong HFS scale, and Cohen’s scale that were used in a large patient study.

### 5.1. Point Rating Scale

The point rating scale is a simple and straightforward method to evaluate the severity of HFS. Patients are asked to rate their spasms on a scale from 1 (mild) to 10 (severe). This scale is easy to use and understand, but it may not capture the full complexity of the condition [15].

### 5.2. Visual Analog Scale (VAS)

The VAS is a measurement instrument that tries to measure a characteristic or attitude that is believed to range across a continuum of values and cannot easily be directly measured. For HFS, patients mark the severity of their spasms on a 10 cm line, with one end representing “no spasms” and the other end representing “worst spasms imaginable”. The VAS can be a useful tool for tracking changes in spasm severity over time [16].

### 5.3. Chong HFS Scale

The Chong HFS scale is a more comprehensive tool that assesses both the frequency and intensity of spasms, as well as their impact on daily activities. The scale ranges from 0 (no spasms) to 4 (severe spasms that significantly interfere with daily activities). This scale provides a more detailed picture of the patient’s condition, but it may be more time-consuming to complete [22].

### 5.4. Cohen’s Scale

Cohen’s scale is a tool specifically designed to assess the impact of HFS on quality of life. It includes items related to physical discomfort, emotional distress, and social and occupational impairment. Scores range from 0 (no impact) to 100 (maximum impact). This scale can provide valuable information about the broader effects of HFS on patients’ lives [23].

Each of these scales has its strengths and weaknesses, and the choice of scale may depend on the specific goals of the assessment. It’s important to consider the patient’s ability to understand and complete the scale, as well as the scale’s ability to capture the aspects of HFS that are most relevant to the patient’s condition and treatment goals.

In addition, the evaluation of improvement is typically conducted through the analysis of pre–post differences which are deemed significant. However, from a methodological perspective, this approach is not entirely adequate, particularly when considering the reliability of the scales or measurements used. Despite this, it is noteworthy that the majority of the literature does not employ “critical differences” grounded in formal reliability scores, or at least, does not establish thresholds for the assessment of improvement.

## 6. Other Uses of BoNT

BoNT has been extensively studied and utilized as a therapeutic agent in various neurological conditions. In the treatment of blepharospasm and oromandibular dystonia, BoNT has been shown to be effective, providing significant relief from symptoms and improving the quality of life for patients [24]. In the context of upper limb spastic paresis, repeated BoNT injections have been found to achieve maximal efficacy, with improvements in muscle tone and motor function observed [25]. Similarly, the use of abobotulinumtoxin A in spastic lower limbs has been investigated in a randomized trial and extension study. The results demonstrated the efficacy and safety of the treatment, with significant reductions in muscle tone and improvements in gait and quality of life [26]. In the realm of music, the rehabilitation of focal hand dystonia in musicians has been explored, with botulinum toxin being a key component of the therapeutic approach [27].

## 7. Side Effects of BoNT

BoNT injections are generally considered safe. However, potential side effects and precautions can include temporary muscle weakness or paralysis in nearby muscles (such as mild facial paresis (23%), diplopia (17%), and ptosis (15%)) [28], pain or bruising at the injection site, headaches, flu-like symptoms, and rarely, more serious systemic effects. Another drawback is the requirement for repeat injections, which might increase the likelihood of developing side effects.

Steps that can be taken to address these side effects include informing the patient about potential side effects and addressing any concerns they might have, monitoring for common side effects such as temporary muscle weakness or drooping and implementing appropriate management strategies if these occur, and assuring patients that most side effects are temporary and typically resolve on their own.

A study by Gentile et al. [29] provides insights into the potential antidotes for botulinum toxin in case of toxicity. The researchers screened a large library of 13,592,287 compounds through a pharmacophore filter, a 3D-QSAR model, and a virtual screening. The compounds with the best affinity were selected, and molecular dynamics simulation studies were conducted on the first four compounds predicted to be the most active. This approach allowed the identification of compounds with a calculated inhibitory activity in the range of 316–500 nM. The study emphasizes the need for an effective antidote to counteract the adverse effects of botulinum toxin, which currently can only be managed by waiting for the pharmacological effect to end. The identified compounds could potentially serve as antidotes to botulinum toxin, providing a promising direction for future research in this area.

## 8. Future Research

BoNT injections can cause discomfort, especially when administered into the face, and the risk of bruising in the eye and facial regions may be undesirable. Consequently, the development of needle-free administration is of considerable interest. However, biological barriers related to the large size of the protein and the precise targeting of muscles present significant challenges [30].

Transdermal drug delivery is currently one of the primary needle-free methods under development. This approach is aimed at relatively superficial delivery and is likely most suitable for treating facial lines and hyperhidrosis [31]. Liquid formulations are also in development for the treatment of glabellar lines, though their applications in neurology are less certain [32].

Hemifacial spasms represent a challenge in neurology due to the lack of a universally standardized or accepted scale for evaluating their severity or progression. This neurological condition, characterized by unilateral involuntary contractions or spasms in facial muscles, is typically diagnosed through individual symptomatology rather than a definitive scoring system. Therefore, the effectiveness of BoNT treatment for HFS varies across different studies, and in some instances, it becomes challenging to objectively demonstrate its impact. The lack of a standardized, objective scale for evaluating hemifacial spasms highlights the need for further research and development in this area. Such a scale could aid in tracking the progression of the condition, evaluating the efficacy of treatments, and improving overall patient care. However, any such scale should also take into account the inherent subjectivity and individual variability present in this condition.

## 9. Conclusions

HFS has the potential to cause interpersonal issues, leading many patients to seek symptom improvement and relief. While surgical treatment can be beneficial in cases with clear indications, when the diagnosis is uncertain or there is a risk associated with surgery, effective symptom improvement can be achieved through BoNT treatment.

## Figures and Tables

**Table 1:** Usual dose of Botulinum toxin.

Muscle	Usual Dose of Injection (Botox)	Usual Dose of Injection (Dysport)
Orbicularis oculi	2.5–5 Unit	7.5–15 Unit
Orbicularis oris	2 Unit	6 Unit
Zygomaticus major	1 Unit	3 Unit
Depressor anguli oris	1–2 Unit	3–6 Unit

Dysport injections dosage using 1:3 or 1:4 Botox/Dysport conversion ratio [13,14].

**Table 2:** Clinical results of large-scale studies.

Author	Patients, N	BoNT Type	Clinical Evaluation	Improvement	Duration of Effect
Park, 1993 [15]	101	Ona	Point rating scale (0–4)	98.4%	16.5 week
Jitpimolmard, 1998 [16]	158	Abo	VAS	97%	3.4 month
Gutierrez, 2021 [19]	162	Ona, Abo	Chong HFS rating scale	78%	Ona: 3.6 month Abo: 3.7 month
Bentivoglio, 2009 [17]	108	Ona, Abo	Subjective assessment	94%	Ona: 105.4 days Abo: 85.4 days
Wu, 2011 [18]	131	Ona	Cohen’s scale	94%	16.5 week

BoNT: botulinum toxin; Abo: Abobotulinumtoxin A; Ona: Onabotulinumtoxin A.

## Data Availability

There is no specific data for this article.
